# Correction: Kaur et al. Synthesis of CaFe_2_O_4_-NGO Nanocomposite for Effective Removal of Heavy Metal Ion and Photocatalytic Degradation of Organic Pollutants. *Nanomaterials* 2021, *11*, 1471

**DOI:** 10.3390/nano15151204

**Published:** 2025-08-06

**Authors:** Manmeet Kaur, Manpreet Kaur, Dhanwinder Singh, Aderbal C. Oliveira, Vijayendra Kumar Garg, Virender K. Sharma

**Affiliations:** 1 Department of Chemistry, Punjab Agricultural University, Ludhiana 141001, Punjab, India; manmeetgill885@gmail.com; 2 Department of Soil Science, Punjab Agricultural University, Ludhiana 141001, Punjab, India; dhanwinder@pau.edu; 3 Institute of Physics, University of Brasilia, Brasilia 70000-000, Brazil; aderbal47@gmail.com (A.C.O.); vijgarg@gmail.com (V.K.G.); 4 Program for Environment and Sustainability, Department of Environmental and Occupational Health, School of Public Health, Texas A&M University (TAMU), College Station, TX 77843-1266, USA

In the original publication [1], there was a mistake in Figure 1 as published. A mistake was made by the first author while compiling the XRD spectra, and the wrong spectrum was overlaid. The overlay error in the figure has been corrected and the updated Figure 1 appears below.

The authors state that the scientific conclusions are unaffected. This correction was approved by the Academic Editor. The original publication has also been updated.

## Figures and Tables

**Figure 1:**
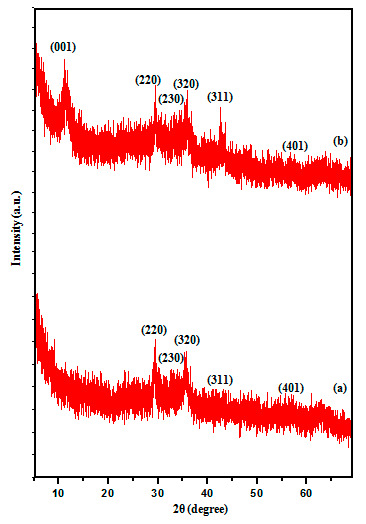
XRD patterns of (**a**) CaFe_2_O_4_ and (**b**) CaFe_2_O_4_-NGO.
